# A brain structural connectivity biomarker for autism spectrum disorder diagnosis in early childhood

**DOI:** 10.1093/psyrad/kkad005

**Published:** 2023-04-20

**Authors:** Xi Jiang, Xiao-Jing Shou, Zhongbo Zhao, Yuzhong Chen, Fan-Chao Meng, Jiao Le, Tian-Jia Song, Xin-Jie Xu, Weitong Guo, Xiaoyan Ke, Xiao-E Cai, Weihua Zhao, Juan Kou, Ran Huo, Ying Liu, Hui-Shu Yuan, Yan Xing, Ji-Sheng Han, Song-Ping Han, Yun Li, Hua Lai, Lan Zhang, Mei-Xiang Jia, Jing Liu, Xuan Liu, Keith M Kendrick, Rong Zhang

**Affiliations:** The Clinical Hospital of Chengdu Brain Science Institute, MOE Key Lab for Neuroinformation, School of Life Science and Technology, University of Electronic Science and Technology of China, Chengdu 611731, China; Neuroscience Research Institute; Key Laboratory for Neuroscience, Ministry of Education of China; Key Laboratory for Neuroscience, National Committee of Health and Family Planning of China; and Department of Neurobiology, School of Basic Medical Sciences, Peking University, Beijing 100191, China; State Key Laboratory of Cognitive Neuroscience and Learning; Beijing Key Laboratory of Brain Imaging and Connectomics; and IDG/McGovern Institute for Brain Research, Beijing Normal University, Beijing 100875, China; The Clinical Hospital of Chengdu Brain Science Institute, MOE Key Lab for Neuroinformation, School of Life Science and Technology, University of Electronic Science and Technology of China, Chengdu 611731, China; The Clinical Hospital of Chengdu Brain Science Institute, MOE Key Lab for Neuroinformation, School of Life Science and Technology, University of Electronic Science and Technology of China, Chengdu 611731, China; Neuroscience Research Institute; Key Laboratory for Neuroscience, Ministry of Education of China; Key Laboratory for Neuroscience, National Committee of Health and Family Planning of China; and Department of Neurobiology, School of Basic Medical Sciences, Peking University, Beijing 100191, China; The Clinical Hospital of Chengdu Brain Science Institute, MOE Key Lab for Neuroinformation, School of Life Science and Technology, University of Electronic Science and Technology of China, Chengdu 611731, China; Chengdu Women's and Children's Central Hospital, School of Medicine, University of Electronic Science and Technology of China, Chengdu 611731, China; Neuroscience Research Institute; Key Laboratory for Neuroscience, Ministry of Education of China; Key Laboratory for Neuroscience, National Committee of Health and Family Planning of China; and Department of Neurobiology, School of Basic Medical Sciences, Peking University, Beijing 100191, China; Neuroscience Research Institute; Key Laboratory for Neuroscience, Ministry of Education of China; Key Laboratory for Neuroscience, National Committee of Health and Family Planning of China; and Department of Neurobiology, School of Basic Medical Sciences, Peking University, Beijing 100191, China; The Clinical Hospital of Chengdu Brain Science Institute, MOE Key Lab for Neuroinformation, School of Life Science and Technology, University of Electronic Science and Technology of China, Chengdu 611731, China; Child Mental Health Research Center, Nanjing Brain Hospital Affiliated of Nanjing Medical University, Nanjing 210029, China; Neuroscience Research Institute; Key Laboratory for Neuroscience, Ministry of Education of China; Key Laboratory for Neuroscience, National Committee of Health and Family Planning of China; and Department of Neurobiology, School of Basic Medical Sciences, Peking University, Beijing 100191, China; The Clinical Hospital of Chengdu Brain Science Institute, MOE Key Lab for Neuroinformation, School of Life Science and Technology, University of Electronic Science and Technology of China, Chengdu 611731, China; The Clinical Hospital of Chengdu Brain Science Institute, MOE Key Lab for Neuroinformation, School of Life Science and Technology, University of Electronic Science and Technology of China, Chengdu 611731, China; Neuroscience Research Institute; Key Laboratory for Neuroscience, Ministry of Education of China; Key Laboratory for Neuroscience, National Committee of Health and Family Planning of China; and Department of Neurobiology, School of Basic Medical Sciences, Peking University, Beijing 100191, China; Radiology Department, Peking University Third Hospital, Beijing 100191, China; Radiology Department, Peking University Third Hospital, Beijing 100191, China; Radiology Department, Peking University Third Hospital, Beijing 100191, China; Department of Pediatrics, Peking University Third Hospital, Beijing 100191, China; Neuroscience Research Institute; Key Laboratory for Neuroscience, Ministry of Education of China; Key Laboratory for Neuroscience, National Committee of Health and Family Planning of China; and Department of Neurobiology, School of Basic Medical Sciences, Peking University, Beijing 100191, China; Wuxi Shenpingxintai Medical Technology Co., Ltd, Wuxi 214091, China; Child Mental Health Research Center, Nanjing Brain Hospital Affiliated of Nanjing Medical University, Nanjing 210029, China; Chengdu Women's and Children's Central Hospital, School of Medicine, University of Electronic Science and Technology of China, Chengdu 611731, China; Chengdu Women's and Children's Central Hospital, School of Medicine, University of Electronic Science and Technology of China, Chengdu 611731, China; Mental Health Institute, Peking University, Key Laboratory of Ministry of Health, The Ministry of Public Health, Beijing 100191, China; Mental Health Institute, Peking University, Key Laboratory of Ministry of Health, The Ministry of Public Health, Beijing 100191, China; Shandong Ke Luo Ni Ke (CLINIC) Medical Technology Co., Ltd, Dezhou 253011, China; The Clinical Hospital of Chengdu Brain Science Institute, MOE Key Lab for Neuroinformation, School of Life Science and Technology, University of Electronic Science and Technology of China, Chengdu 611731, China; Neuroscience Research Institute; Key Laboratory for Neuroscience, Ministry of Education of China; Key Laboratory for Neuroscience, National Committee of Health and Family Planning of China; and Department of Neurobiology, School of Basic Medical Sciences, Peking University, Beijing 100191, China; Autism Research Center of Peking University Health Science Center, Beijing 100191, China; Department of Integration of Chinese and Western Medicine, School of Basic Medical Sciences, Peking University, 100191,Beijing, China

**Keywords:** autism spectrum disorder, diffusion tensor imaging, fractional anisotropy, brain structural connectivity, individual diagnosis, early childhood

## Abstract

**Background:**

Autism spectrum disorder (ASD) is associated with altered brain development, but it is unclear which specific structural changes may serve as potential diagnostic markers, particularly in young children at the age when symptoms become fully established. Furthermore, such brain markers need to meet the requirements of precision medicine and be accurate in aiding diagnosis at an individual rather than only a group level.

**Objective:**

This study aimed to identify and model brain-wide differences in structural connectivity using diffusion tensor imaging (DTI) in young ASD and typically developing (TD) children.

**Methods:**

A discovery cohort including 93 ASD and 26 TD children and two independent validation cohorts including 12 ASD and 9 TD children from three different cities in China were included. Brain-wide (294 regions) structural connectivity was measured using DTI (fractional anisotropy, FA) together with symptom severity and cognitive development. A connection matrix was constructed for each child for comparisons between ASD and TD groups. Pattern classification was performed on the discovery dataset and the resulting model was tested on the two independent validation datasets.

**Results:**

Thirty-three structural connections showed increased FA in ASD compared to TD children and associated with both autistic symptom severity and impaired general cognitive development. The majority (29/33) involved the frontal lobe and comprised five different networks with functional relevance to default mode, motor control, social recognition, language and reward. Overall, classification achieved very high accuracy of 96.77% in the discovery dataset, and 91.67% and 88.89% in the two independent validation datasets.

**Conclusions:**

Identified structural connectivity differences primarily involving the frontal cortex can very accurately distinguish novel individual ASD from TD children and may therefore represent a robust early brain biomarker which can address the requirements of precision medicine.

## Introduction

There is an increasing consensus that children with autism spectrum disorder (ASD) have an aberrant pattern of brain development (Hazlett *et al*., 2011; Girault and Piven, 2020). A number of structural magnetic resonance imaging studies using diffusion tensor imaging (DTI) have identified altered fractional anisotropy (FA), which is a widely used index in DTI reflecting the integrity of axonal density and myelination, in white matter tracts of ASD, particularly between the frontal and occipital lobes (Andrews *et al*., 2019; Ingalhalikar *et al*., 2011; Walker *et al*., 2012;), and in corpus callosum (Adluru *et al*., 2009; Andrews *et al*., 2019; Catani *et al*., 2016; Di *et al*., 2018; Ingalhalikar *et al*., 2011; Koshiyama *et al*., 2020; Solso *et al*., 2016; Walker *et al*., 2012). These tract-based studies reflect that the developmental changes in FA occur with increases in fiber tracts occurring during the first few years, followed by decreases in later childhood and adolescence, through into adulthood (Andrews *et al*., 2019; Catani *et al*., 2016; Koshiyama *et al*., 2020; Walker *et al*., 2012). Moreover, the discrimination accuracies between ASD and typically developing (TD) individuals using the tract-based features and following machine learning classification approaches have achieved modest performance (71–85% classification accuracy) in previous studies (Adluru *et al*., 2009; ElNakieb *et al*., 2019; Ingalhalikar *et al*., 2011; Zhang *et al*., 2016, 2018) and have not included validation using independent datasets. Besides tract-based analysis, there is also increasing interest in assessing the altered structural connectivity between distinct brain regions, which reflects in greater detail the interactions within large-scale brain networks, in ASD (Cai *et al*., 2022; Fishman *et al*., 2015; Li *et al*., 2018). To date only one small study has reported increased structural connectivity in young preschool children with ASD in a small network of 12 frontal, temporal and occipital regions (Li *et al*., 2018). However, it is largely unknown how the identified structural connectivity features can achieve satisfying classification performance between ASD and TD across independent datasets to adequately address the aims of precision medicine (Quinlan *et al*., 2020) to be able to accurately discriminate at the level of the individual between ASD and TD.

In the current study we have therefore used DTI to identify differences in inter-regional structural connectivity at the whole-brain level in ASD compared to TD children in 294 different brain regions. We chose to restrict the age range of children to 3.5–6 years old since this corresponds to the period when ASD symptoms have become robustly established (Lord *et al*., 2018), the average age for an initial diagnosis (3.6 years–van't Hof *et al*., 2021) and in studies investigating the effects of behavioral interventions (4.5 years–Sandbank *et al*., 2020). Previous tractography-based research also suggests that at this age overall increased structural connectivity of the main fiber tracts in the frontal lobe of ASD and TD children may be less pronounced (Solso *et al*., 2016). In order to address the aims of precision medicine that brain markers should be accurate at discriminating disorders at an individual as opposed to group level (Peterson, 2020), we not only used a large discovery dataset of ASD and TD children to establish features resulting in a satisfactory discrimination accuracy between groups but also assessed the ability of these established features to reliably identify individuals with ASD as opposed to TD in two independent datasets.

Based on previous studies, we firstly hypothesized that ASD children would exhibit significantly greater FA in structural connections at the whole brain level and particularly involving frontal regions compared to TD children. Secondly, we hypothesized that altered structural connections would be in networks associated with ASD symptoms and cognitive and behavioral development. Finally, we hypothesized that the identified structural connectivity changes would accurately predict ASD diagnosis at the individual level not only within the original discovery dataset but also in two independent validation datasets.

## Methods and Materials

### Participants

The present study included three independent datasets: a discovery and two validation datasets. This cross-sectional study took place from 2013 to 2020 for participant recruitment and data acquisition, and in 2021 for data analysis, and involved a discovery cohort and two independent validation cohorts from three different cities in China. All procedures comply with the Helsinki Declaration.

#### Discovery dataset (Beijing)

The experiment was approved by the ethics committee of the Peking University Institutional Review Board (approval no. IRB00001052-13079). A total of 119 pre-school children either diagnosed with ASD (n = 93) or TD children (n = 26) were recruited. The age range of participants was 3.5 to 6 years, which is regarded as the time of the most severe emerging symptoms of autism (Lord *et al*., 2018). Children with ASD were recruited through pediatric psychiatric clinics and autism rehabilitation training centers in Beijing. Age and gender matched TD children were also recruited through online social platforms or day care centers in Beijing.

#### ASD validation dataset (Chengdu)

The experiment was approved by the ethics committee of the University of Electronic Science and Technology of China (approval no. 1420190601). A total of 12 ASD children were recruited aged 3 to 8 years through the child healthcare department of Chengdu Women's and Children's Central Hospital.

#### TD validation dataset (Nanjing)

The experiment was approved by the medical ethics committee of the Brain Hospital affiliated to Nanjing Medical University (approval no. KY043). A total of 9 TD children were recruited aged 4 to 6 years either through the Nanjing child mental health research center, online social platforms or day care centers.

All the participants’ parents in the three datasets were informed in detail of the research objectives and procedures, and provided written informed consents. Exclusion criteria were: (i) neurological complications, such as epilepsy, cerebral palsy, Fragile X syndrome etc. which are provided by previous clinical data or diagnosis. Brain images were reviewed by neuroradiologists to confirm absence of neurological abnormalities. (ii) medical intervention, such as antipsychotic drugs, transcranial magnetic stimulation, acupuncture etc.; (iii) diagnostic imaging anomalies or craniocerebral trauma; (iv) other contraindications to MRI; (v) TD children had no family histories of any mental disorders and exhibited no evidence of developmental delay.

### Clinical diagnosis

Participants in ASD groups were diagnosed at either Peking University Sixth Hospital or Beijing Children's Hospital, Beijing, China for Beijing dataset, or at Chengdu Women's and Children's Central Hospital, Chengdu, China for Chengdu dataset. All children in the ASD group met the diagnostic criteria of Diagnostic and Statistical Manual of Mental Disorders IV-Text Revision (DSM-IV-TR) (American Psychiatric Association, 2000) or Fifth Edition (DSM-5) (American Psychiatric Association, 2013) and International Statistical Classification of Diseases and Related Health Problems 10th revision (ICD-10) (World Health Organization, 2004). In addition, ASD diagnosis was confirmed in the Beijing dataset using the Autism Diagnostic Observation Schedule (ADOS) (Lord *et al*., 1989) Traditional Mandarin version, module 1 or module 2 based on the child's language ability. For children in the Chengdu dataset, diagnosis was confirmed using ADOS-2 (Lord *et al*., 2008). Moreover, in the Beijing ASD and TD cohorts, cognitive and behavioral development was also assessed using the Gesell Developmental Scale (GDS) (Jin *et al*., 2007) administered by an experienced pediatrician. The GDS is a measure of cognitive and behavioral development and adaptability including five components (gross motor, fine motor, adaptive, language and personal social behaviors).

### MRI acquisition and preprocessing

Children in both ASD and TD groups of the three datasets were sedated by oral administration of chloral hydrate at the 50 mg/kg body weight (1 g maximum dose), commonly used for pediatric clinical imaging. During the MRI scan, children wore earplugs and de-noising headsets to reduce the noise, and parents were encouraged to remain in the scanning room to ensure safety in case the child awoke.

For the discovery dataset MRI images were acquired on a GE 3T MR750 scanner with a 12-channel head coil at the Peking University Third Hospital. DTI data were obtained with an echo-planar imaging sequence: TR = 9 000 ms, TE = 89.4 ms, FOV = 256 mm, matrix size = 128 × 128, voxel size = 2 mm isotropic, 75 slices covering the whole brain with no gap, 32 diffusion directions, b-value = 1 000 s/mm^2^.

For the ASD validation dataset MRI images were acquired on a GE 3T MR750 scanner with an 8-channel head coil at the University of Electronic Science and Technology of China. DTI data were acquired with an echo-planar imaging sequence: TR = 8 500 ms, FOV = 256 mm, matrix size = 128 × 128, voxel size = 2 mm isotropic, 60 slices covering the whole brain with no gap, 32 diffusion directions, and b-value = 1 000 s/mm^2^.

For the TD validation dataset MRI images were acquired on a 3T Verio MRI system (Siemens Medical Systems, Germany) with a birdcage gradient head coil at Nanjing Brain Hospital. DTI data were scanned with a single-shot echo-planar sequence: TR = 9 000 ms, TE = 104 ms, flip angle = 90°, FOV = 230 mm, matrix size = 128 × 128, voxel size = 1.8 mm × 1.8 mm × 2.5 mm, 60 slices covering the whole brain with no gap, NEX = 2.0, 32 diffusion directions, and b-value = 1 000 s/mm².

Pre-processing for DTI included skull removal, motion correction, eddy correction, and tensor fitting via FSL (Jenkinson *et al*., 2012). Specifically, the most widely used FA (fractional anisotropy) measure in DTI was adopted in this study. Deterministic streamline fibers were then reconstructed from the pre-processed DTI based on the diffusion tensor model via DSI Studio (Yeh *et al*., 2013). The major fiber tracking parameters were the same as in previous studies (Zhang *et al*., 2020): fiber count = 40 000, step size = 1 mm, max turning angle = 60°, minimum fiber length = 30 mm, maximum fiber length = 300 mm, smoothing = 1.

### Overview of analysis

**Figure 1:**
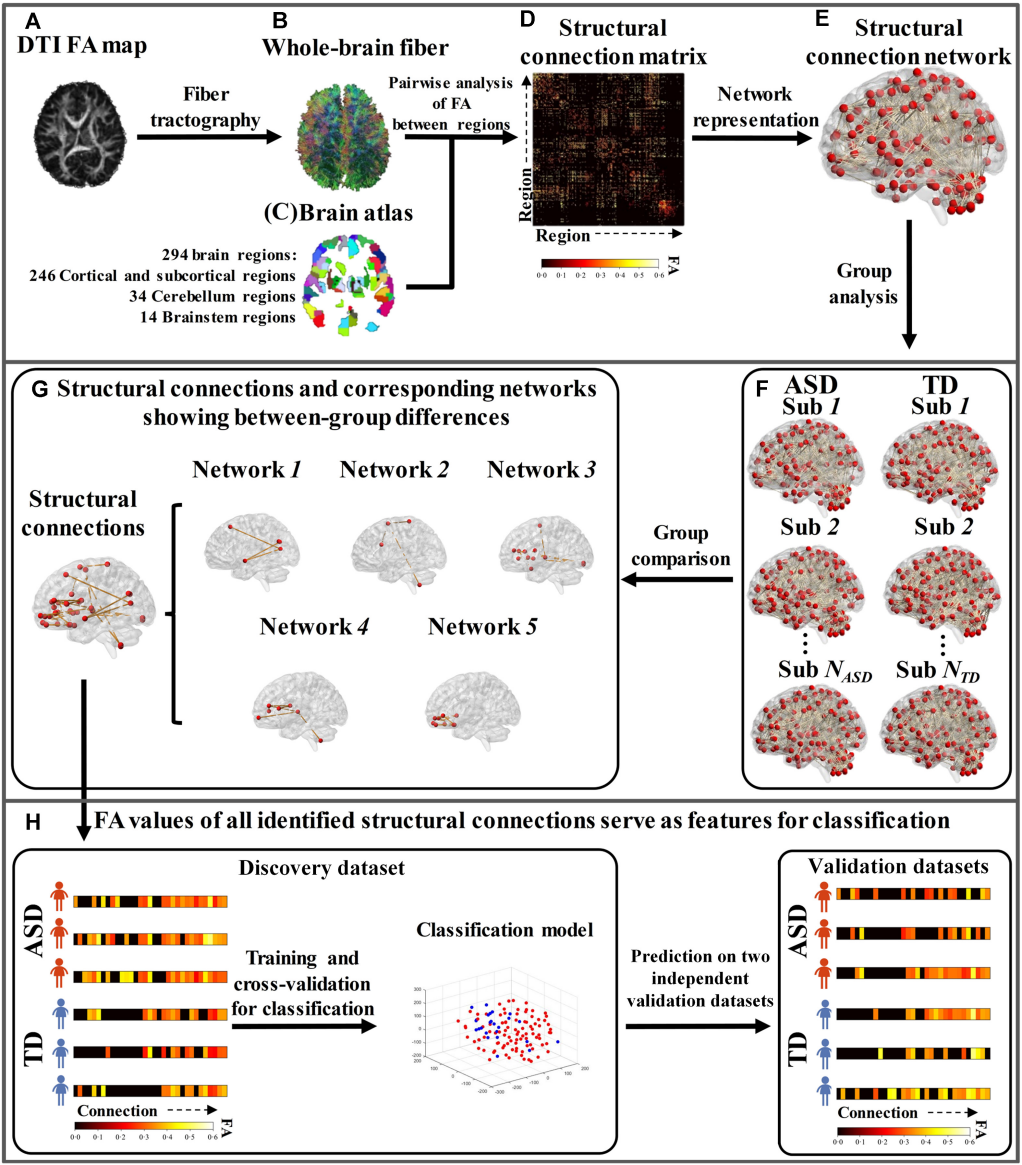
Flow chart of ASD identification framework. (**A**) The pre-processed DTI FA map. (**B**) Reconstructed whole-brain streamline fibers. (**C**) Young children's brain atlas. (**D**) Structural connection matrix for each participant. (**E**) The individual structural connection network. (**F**) All individual structural connection networks in ASD and TD groups. (**G**) The structural connections and associated networks showing differences between ASD and TD groups. (**H**) Pattern classification between ASD and TD using all structural connections showing between-group differences.

The flow chart of ASD identification framework is illustrated in Fig. 1. Based on the pre-processed DTI FA map (Fig. 1(A)), the whole-brain streamline fibers were reconstructed (Fig. 1(B)) and adopted to the brain atlas (Fig. 1(C)). To construct the structural connection matrix for each subject (Fig. 1(D)), we calculated the pair-wise FA value between every two brain regions. The constructed structural connection matrix was further projected into the brain for each participant (Fig. 1(E)). Group comparisons were then performed based on all structural connection networks between ASD and TD groups (Fig. 1(F)) to obtain the between-group differences (Fig. 1(G)). The resulting connections were finally adopted as features to perform pattern classification between ASD and TD groups (Fig. 1(H)). The details of each step are demonstrated in the following sections.

### Construction of structural connection network

The brain atlas used in this study included 294 non-overlapping brain regions consisting of 246 cortical and subcortical regions, 34 cerebellar regions, and 14 brainstem regions (Fig. 1(C)). The cortical and subcortical regions were from the Brainnetome Atlas (Fan *et al*., 2016) with 123 homotopic regions in each hemisphere; cerebellar regions were from the Human Cerebellar Probabilistic Magnetic Resonance Atlas (Diedrichsen *et al*., 2009) and brainstem regions were from the Human Brainstem Standard Neuroanatomy Atlas (Edlow *et al*., 2012). Since the three atlases were originally defined in the adult MNI152 standard space, we first performed linear registration to warp the T1-weighted adult MNI152 image to the space of T1-weighted template image of children aged 4.5–8.5 years (https://www.mcgill.ca/bic/software/tools-data-analysis/anatomical-mri/atlases/nihpd) (Fonov *et al*., 2011), and then applied the linear transformation to the brain atlas to warp it to the children's template image space as well in order to obtain the young children's brain atlas.

The reconstructed whole-brain streamline fibers in each child (Fig. 1(B)) were then aligned to the young children's brain atlas space via DSI Studio (Yeh *et al*., 2013). The structural connection matrix for each child (Fig. 1(D)) was obtained by calculating the pair-wise mean FA value between every two brain regions via DSI Studio (Yeh *et al*., 2013), and further represented as a structural connection network (Fig. 1(E)) in which the nodes were the brain regions and edges were the mean FA values between two nodes.

### Identification of structural connections showing differences between ASD and TD

Based on the structural connection networks of all children in the ASD and TD groups (Fig. 1(F)), we adopted the widely used Network-based Statistic (NBS) approach (Zalesky *et al*., 2010) to identify the structural connections and associated networks showing between-group differences (Fig. 1(G)). As a non-parametric statistical method, NBS first performs a large number of univariate hypothesis tests on all edges in the network, then clustering-based statistics, and finally permutation tests to calculate the family-wise error rate (FWER) corrected p-values for each sub-network consisting of edges with group differences. We adopted the NBS Connectome toolbox (Zalesky *et al*., 2010) implemented in Matlab to perform the analysis. The structural connection networks of all participants in ASD and TD groups were the inputs with gender and age as covariates. Next we performed the NBS analysis to identify both increased and decreased FA values of structural connections and associated networks in ASD compared to TD.

### Pattern classification between ASD and TD children

Based on the identified structural connections showing between-group differences (Fig. 1(G)), we further adopted FA values of those connections as features to perform pattern classification between the ASD and TD groups. We used the discovery dataset as the training dataset to establish the classification model. For training and leave-one-out cross-validation, we employed the widely used support vector machine (SVM) approach. The training model was then applied to the two independent validation datasets to validate its generalizability in terms of accuracy, sensitivity, and specificity. Specifically, we adopted the widely used cost-support vector classification (C-SVC), and the radial basis function (RBF) as the kernel function in SVM. The optimal values of parameters c (i.e. cost, c ranged from 15 to 16) and g (i.e. gamma, g ranged from 0.08 to 1) in RBF were obtained by a hyperparameter optimization framework of optuna (Akiba *et al*., 2019).

### Potential effect of imbalanced sample size between ASD and TD

In view of the imbalanced group sample sizes in the discovery dataset (93 ASD and 26 TD), we adopted both up-sampling and down-sampling approaches to alleviate the potential model overfitting as well as classification bias problem. The first approach was the well-known SMOTE (Synthetic Minority Oversampling Technique algorithm) which upsampled the minority group of data samples by taking random simple replication of nearest neighbour samples to achieve balance between the two groups (Chawla, Bowyer *et al*., 2002). In this study, we upsampled the TD sample size to 93 to match the ASD sample size. The second approach was down-sampling. Each time we randomly selected 26 out of 93 samples from the ASD group to train the classification model together with all 26 TD samples, and repeated the procedure 1 000 times to obtain the averaged classification accuracy. Both approaches confirmed that our classification model was not influenced by the imbalanced sample sizes.

### Statistical analysis

Independent two-sample t-tests were utilized to identify the different structural connections between ASD and TD (n = 10 000 permutation times, significance level *P* < 0.05, FWE corrected) in NBS. Independent sample t-tests were used for continuous variables including age, BMI, head circumference, GDS total score, and FA between ASD and TD. Chi-square tests were used for categorical variables including gender and handedness between ASD and TD. Pearson's linear correlation coefficients were computed between the averaged FA value and ADOS and GDS scores (one-sample t-tests, significance level at *P* < 0.05, FDR corrected). A mediation model was conducted (PROCESS) (Hayes, 2012) using bootstrap analysis to investigate the relationship between the averaged FA value, ADOS total and GDS total scores (bootstrap = 1 000).

## Results

### Subject demographics and behavioral measures

Table 1 summarizes demographic and other information for ASD and TD groups in the different datasets and ADOS scores for ASD children. There were no group differences in age, gender, BMI, handedness, or head circumference in the discovery dataset, although as expected the total GDS score was significantly less in the ASD group indicating impaired cognitive and behavioral development.

**Table 1:** Subject: demographics and behavioral measures of the three datasets.

	TD		ASD		Statistics (t or χ2)	Significance (p)
	N	Mean (SD)	N	Mean (SD)		
**Discovery (Beijing) dataset**						
Age, years	26	4.70 (0.46)	93	4.58 (0.55)	−1.07^a^	0.29
Gender (boys: girls)	26	20:6	93	85:8	2.83^b^	0.09
BMI	26	15.64 (1.68)	76	16.03 (1.77)	0.87^a^	0.39
Handedness (R: D: L)	26	20:6:0	90	78:9:3	3.76^b^	0.15
Head Circumference, cm	26	51.24 (1.48)	90	51.79 (1.72)	1.49^a^	0.14
ADOS social interaction sub-scale score			86	9.88 (2.54)		
ADOS communication sub-scale score			86	5.57 (1.87)		
ADOS total score (social interaction + communication)			86	15.45 (4.02)		
GDS total score	26	94.39 (10.1)	82	64.34 (17.1)	8.50^a^	<0.001
**ASD validation (Chengdu) dataset**						
Age, years			12	4.98 (1.22)		
Gender (boys: girls)			12	11:1		
ADOS-2 total score (social affect + restricted and repetitive behavior)			12	18.58 (4.46)		
**TD validation (Nanjing) dataset**						
Age, years	9	5.44 (0.84)				
Gender (boys: girls)	9	6:3				

Abbreviations: BMI = body mass index; R = right handed; D = double handed; L = left handed; SD = standard deviations.

a Independent two-sample t test, t score;

b Chi-square test, χ2.

### Increased FA connections and networks in ASD compared to TD children

**Figure 2:**
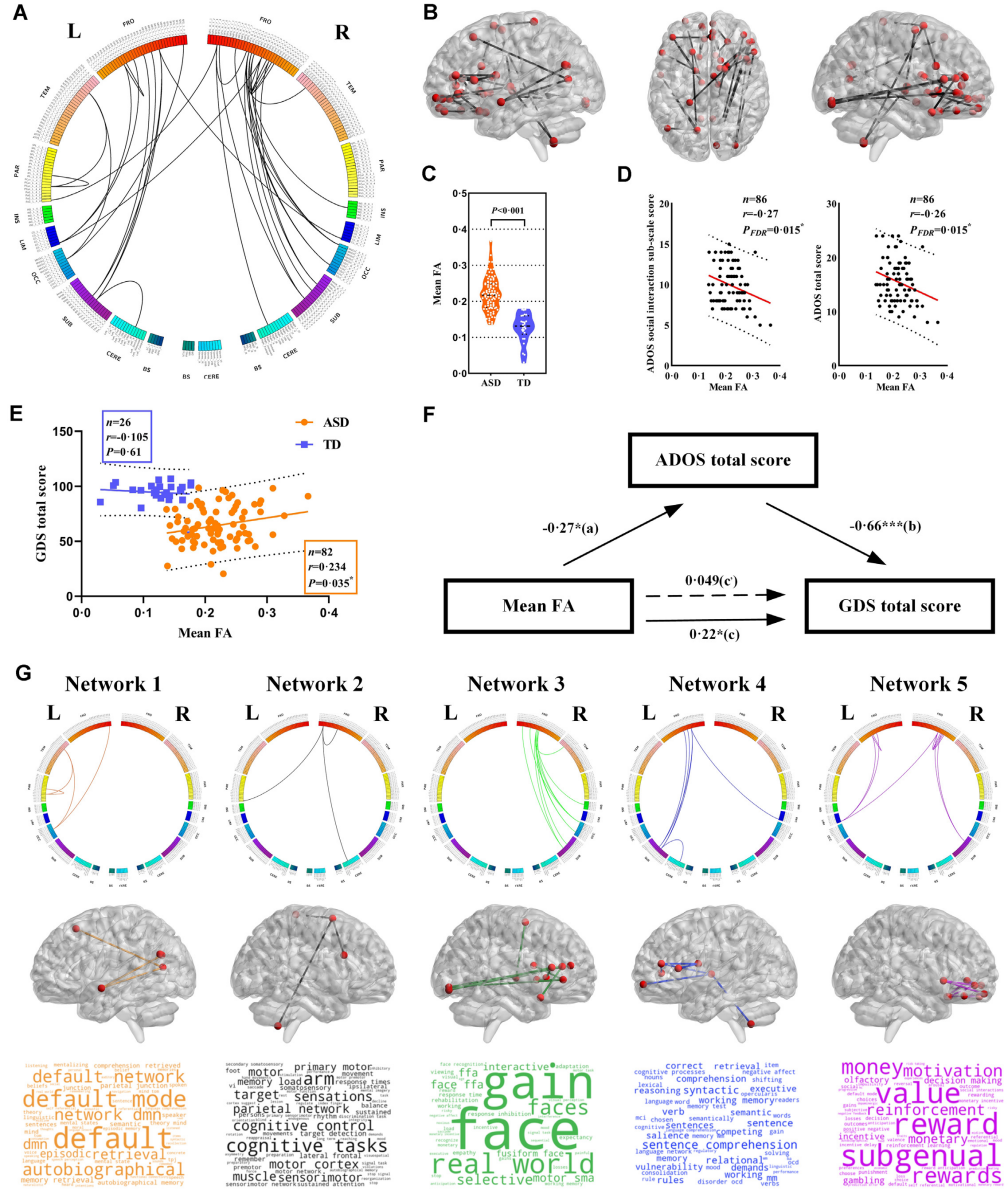
Increased FA connections and networks in ASD compared to TD children. (**A**) The 33 increased FA values of structural connections in ASD in circos plot. Abbreviations: L, left hemisphere; R, right hemisphere; FRO, frontal lobe; TEM, temporal lobe; PAR, parietal lobe; INS, insular lobe; LIM, limbic lobe; OCC, occipital lobe; SUB, subcortical nuclei; CERE, cerebellum; BS, brain stem. (**B**) Locations of 33 connections on the cortical surface. (**C**) Averaged FA value of 33 connections in ASD and TD groups. (**D**) Correlations between averaged FA value of the 33 connections in ASD and ADOS ‘social interaction’ sub-scale score and total (‘communication’ + ‘social interaction’) score. (**E**) Correlations between averaged FA value of the 33 connections and ADOS social communication and total scores and GDS total score. (**F**) Mediation analysis between averaged FA, GDS total score and ADOS total score (path a = −0.27, *P* = 0.018; path b = −0.66, *P* < 0.001; path c = 0.22, *P* = 0.047; path ${{\mathrm{c}}}^,$= 0.049, *P* = 0.58). ( **G**) The 33 increased connections were further categorized into 5 structural networks. In each network, row 1–3 shows the structural connections in circos plot, locations of the connections on cortical surface, and the word cloud of functional annotation via meta-analysis respectively.

We identified 33 increased but no decreased FA values of structural connections in ASD compared to TD children (Figs. 2(A) and 2(C), Table 2). Notably, 29 out of 33 connections were associated with the frontal lobe. Moreover, 30 out of 33 connections were intra-hemispheric. Figure 2(B) illustrates the locations of 33 connections on the cortical surface. In the ASD group averaged FA values were significantly negatively correlated (FDR corrected) with ADOS total and ‘social interaction’ sub-scale scores (Fig. 2(D)) and positively correlated with the GDS total score (Fig. 2(E)). In the TD group there was a slight but not significant negative correlation between averaged FA values and GDS score (Fig. 2(E)). A mediation analysis indicated that within the ASD group the ADOS total score was the main mediator of the effects on the averaged FA value and GDS total score (Fig. 2(F)).

**Table 2:** Profiles: of the 33 structural connections with increased FA in children with ASD.

			MNI coordinates				MNI coordinates		
No.	Network	Region name	X	Y	Z	Region name	X	Y	Z
**1**	**1**	STG_L	−55	−3	−10	PCun_L	−12	−67	25
**2**		IPL_L	−47	−65	26	PCun_L	−12	−67	25
**3**		SFG_L	−18	24	53	MVOcC _L	−13	−68	12
**4**		STG_L	−55	−3	−10	MVOcC _L	−13	−68	12
**5**	**2**	SFG_R	20	4	64	IFG_R	45	16	25
**6**		SFG_R	20	4	64	PoG_L	−21	−35	68
**7**		SFG_R	20	4	64	Right_IX	6	−54	−50
**8**	**3**	IFG_R	48	35	13	LOcC_R	32	−85	−12
**9**		IFG_R	54	24	12	LOcC_R	32	−85	−12
**10**		MFG_R	42	44	14	MVOcC_R	10	−85	−9
**11**		IFG_R	48	35	13	MVOcC_R	10	−85	−9
**12**		IFG_R	51	36	−1	MVOcC_R	10	−85	−9
**13**		IFG_R	51	36	−1	BG_R	22	8	−1
**14**		IFG_R	51	36	−1	BG_R	14	5	14
**15**		IFG_R	48	35	13	STG_R	47	12	−20
**16**		IFG_R	54	24	12	INS_R	36	18	1
**17**		SFG_R	7	−4	60	Tha_R	12	−14	1
**18**		IFG_R	48	35	13	Tha_R	12	−14	1
**19**	**4**	MFG_L	−41	41	16	BG_L	−14	2	16
**20**		MFG_L	−41	41	16	Tha_L	−7	−12	5
**21**		IFG_L	−53	23	11	BG_L	−14	2	16
**22**		MFG_L	−41	41	16	CG_R	5	41	6
**23**		MFG_L	−26	60	−6	Tha_L	−7	−12	5
**24**		Tha_L	−7	−12	5	Left_IX	−8	−54	−48
**25**	**5**	OrG_R	23	36	−18	OrG_R	6	57	−16
**26**		OrG_R	23	36	−18	OrG_R	9	20	−19
**27**		OrG_L	−6	52	−19	OrG_L	−10	18	−19
**28**		OrG_R	6	47	−7	OrG_R	9	20	−19
**29**		OrG_L	−7	54	−7	CG_L	−4	39	−2
**30**		OrG_R	6	47	−7	CG_L	−4	39	−2
**31**		OrG_L	−10	18	−19	CG_L	−4	39	−2
**32**		OrG_R	6	47	−7	BG_R	15	14	−2
**33**		OrG_R	6	57	−16	BG_R	15	14	−2

Abbreviations: L = left; R = right; STG = superior temporal gyrus; PCun = precuneus; IPL = inferior parietal lobule; SFG = superior frontal gyrus; MVOcC = medioventral occipital cortex; IFG = inferior frontal gyrus; PoG = postcentral gyrus; LOcC = lateral occipital cortex; Tha = thalamus; MFG = middle frontal gyrus; OrG = orbitofrontal gyrus; CG = cingulate gyrus; BG = basal ganglia; INS = insula.

Connections with increased FA were further categorized into 5 structural networks via NBS (Fig. 2(G) and Table 2) together with a functional characterization using the Neurosynth platform, and visualized as a word cloud. Network 1: default mode and memory retrieval functions, including: left superior temporal gyrus, precuneus, inferior parietal lobule, superior frontal gyrus, and medioventral occipital cortex; Network 2: motor function, including: right superior and inferior frontal gyri, right cerebellum lobe IX and left postcentral gyrus; Network 3: visual and facial recognition functions, including: right inferior and superior frontal gyri, lateral occipital cortex, pre-motor thalamus, middle frontal gyrus, medioventral occipital cortex, basal ganglia, superior temporal gyrus and insula; Network 4: language and cognitive functions, including: left middle and inferior frontal gyri, thalamus, cingulate gyrus, basal ganglia and cerebellum lobe IX; Network 5: social and general reward functions, including: bilateral orbitofrontal and cingulate gyri and basal ganglia.

### Classification accuracy between ASD and TD children

**Figure 3:**
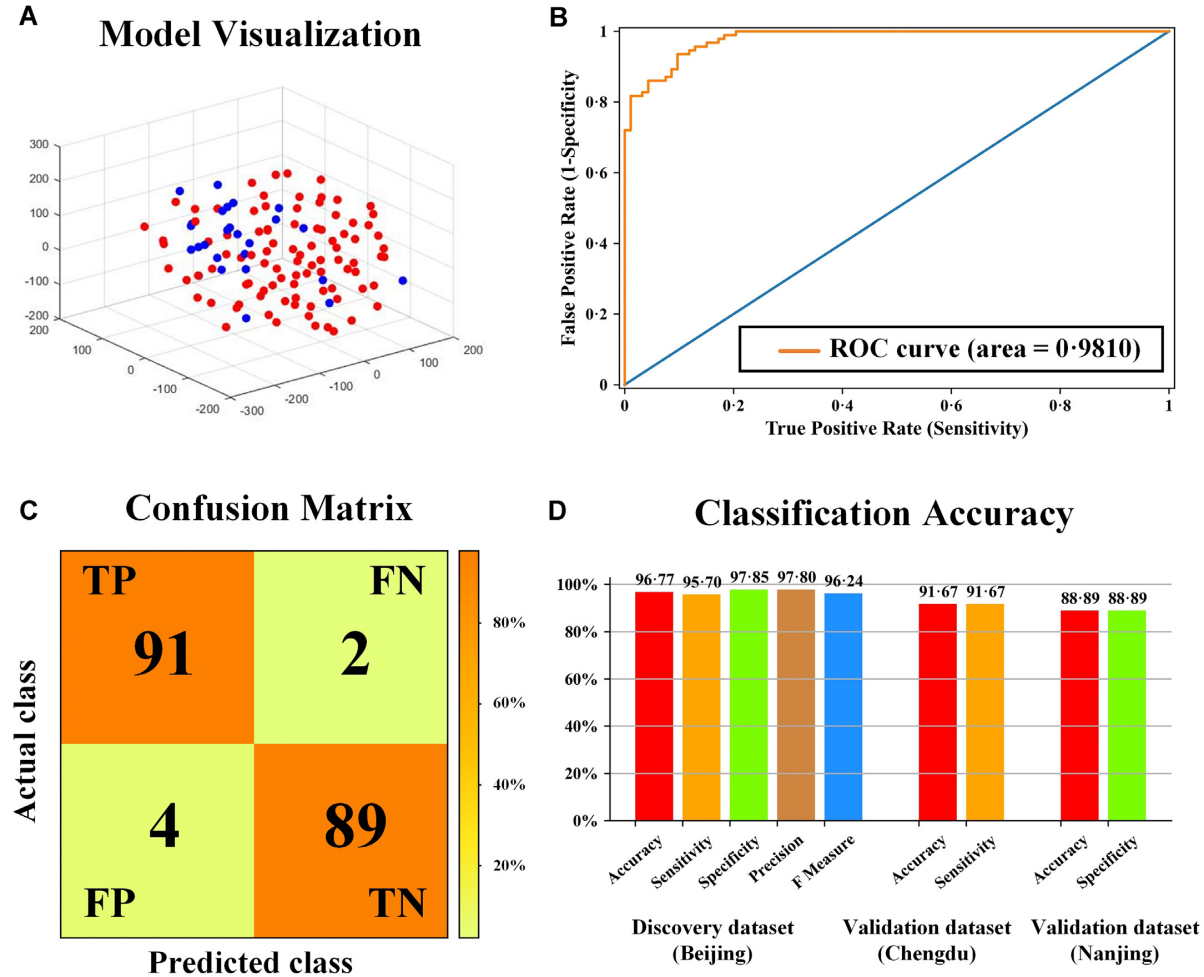
Classification Accuracy between ASD and TD. (**A**) Classification model in a 3-dimensional feature space after performing dimensionality reduction using the t-distributed stochastic neighbor embedding (t-SNE) algorithm. Red and blue dots represent ASD and TD subjects, respectively. (**B**) The Receiver Operating Characteristic (ROC) curve of the training model. (**C**) The confusion matrix of the training model. The colorbar represents the proportion of correctly classified subjects among all subjects. (**D**) The detailed classification accuracy metrics including accuracy, sensitivity, specificity, precision, and F measure in both discovery and validation datasets.

We first up-sampled the discovery dataset to 186 subjects with 93 ASD and 93 TD and trained the classification model in a 33-dimensional feature space based on the 33 connections using a leave-one-out cross-validation strategy. Figure 3(A) shows the classification model in a 3-dimensional feature space after performing dimensionality reduction using the t-distributed stochastic neighbor embedding (t-SNE) algorithm (Van and Hinton, 2008). The Receiver Operating Characteristic (ROC) curve of the training model is shown in Fig. 3(B). The area under the ROC curve (AUC) was 0.981, indicating the robustness of the training model. The confusion matrix of the training model is shown in Fig.   3(C). Accuracy, sensitivity, specificity, precision, and F measure in both discovery and validation datasets are reported in Fig. 3(D) with the proposed model achieving high classification accuracy in both discovery (96.77%) and independent validation datasets (91.67% and 88.89%). The alternative down-sampling strategy of the discovery dataset by 1 000 times also achieved high classification accuracy in both discovery (94.85± 1.30%) and independent validation datasets (91.63± 5.55% and 80.04± 5.52%). Thus overall, the classification model showed both high accuracy and generalizability for ASD identification across different datasets without being influenced by the imbalanced sample sizes of the discovery dataset.

## Discussion

Our findings have revealed the presence of a small number of inter-regional structural connections within the brains of young children with ASD which exhibit increased FA compared to TD and negatively associated with symptom severity. The majority of affected regional connections involve the frontal cortex and overall they achieved a classification accuracy of 96.77% for discriminating between ASD and TD individuals in the discovery dataset. Importantly in the context of the requirements for biomarkers in precision medicine, these affected regional connections were also reliable in discriminating ASD or TD individuals (91.67% and 88.89%) in two small independent datasets. The 33 inter-regional structural connections could be clustered into 5 independent networks with relevance to a range of behavioral functions influenced in ASD.

In support of our original hypothesis, the majority of the 33 structural connections showing increased FA in children with ASD in the current study involved the frontal lobe including intrinsic short range frontal-frontal connections and longer range frontal-occipital, frontal-thalamic and frontal-limbic ones. This is in agreement with findings from other studies (Middleton and Strick, 2001; Casanova, 2007; Johnson *et al*., 2014; Catani *et al*., 2016; Solso *et al*., 2016) and supports the conclusion that alterations in both intrinsic and extrinsic frontal lobe structural connectivity contribute fundamentally to ASD.

The altered structural connections in children with ASD could be clustered into 5 individual networks encompassing default mode, motor, visual and facial recognition, language comprehension and memory and reward functions. The largest single frontal lobe cluster (network 5) involved orbitofrontal regions and their connections with the basal ganglia. These intrinsic frontal connections are strongly associated with social and other types of reward processing as well as decision making (Kringelbach, 2005) and these functions are known to be impaired in ASD (Scott-Van *et al*., 2010; Jin *et al*., 2020). A second large network (network 3) included right inferior and superior frontal gyri, lateral occipital cortex, pre-motor thalamus, middle frontal gyrus, medioventral occipital cortex, basal ganglia, superior temporal gyrus and insula involved in visual and facial recognition functions impaired in ASD (Vlamings *et al*., 2010) and is similar to one reported to have increased structural connectivity in a smaller number of pre-school children with ASD (Li *et al*., 2018). Two other clusters (networks 2 and 4) involved inferior, medial and superior frontal gyri connections with thalamus, basal ganglia, cingulate, insula, occipital cortex, post-central gyrus and cerebellum associated with social cognition, language comprehension, sensory and motor processing functions (Fuster, 2001; Turken & Dronkers, 2011; Badre and Nee, 2018), all of which are impaired in ASD (Vlamings *et al*., 2010; Kjellmer *et al*., 2012; Harrison *et al*., 2021; Lim *et al*., 2021). The remaining cluster (network 1) primarily involved connections between the superior temporal gyrus and inferior parietal lobule with the precuneus in the default mode network, associated with self-processing, experience of agency, autobiographic and episodic memory retrieval and visuospatial imagery. Default mode dysfunction has been consistently reported in ASD (Lynch *et al*., 2013) as well as impaired self-processing, sense of agency, autobiographical and episodic memory (Souchay *et al*., 2018; Zhao *et al*., 2013).

A previous study using DTI measures and classification techniques to identify ASD compared to TD children employed shape representations of white matter fiber tracts as features, and achieved 75.34% discrimination accuracy using a leave-one-out cross-validation approach (Adluru *et al*., 2009). Other studies have adopted the anisotropy scores of regions of interest as features, and achieved 80% (Ingalhalikar *et al*., 2011) or 71% (ElNakieb *et al*., 2019) accuracy using leave-one-out cross-validation. In our current study, we adopted the DTI-derived FA values of structural connections as features, and achieved a much higher classification accuracy in both the discovery dataset (96.77%) and, importantly, in independent validation datasets (91.67% and 88.89%), demonstrating satisfying classification and generalization ability of our model across different datasets and at individual level.

Unexpectedly, we found a significant negative correlation between the averaged FA values of the 33 altered connections in children with ASD and ADOS total and social sub-scale scores, indicating that symptom severity was actually lower in children with greater FA. Scores on GDS were also positively correlated with FA values in the ASD group but slightly negatively correlated in the TD group. A mediation analysis identified that ADOS scores were primarily mediating both FA values and GDS scores in the ASD group. This may indicate an experience-dependent compensatory effect is occurring in children with ASD whereby increased FA contributes to reduced symptom severity and enhanced cognitive and behavioral development. A social experience compensation effect has previously been described in behavioral studies of autism (Livingston *et al*., 2020). Interestingly, a tractography study has reported a positive association between increased frontal lobe FA and symptom severity in very young children but a negative one in older children in the age-range of the current study (Solso *et al*., 2016). Thus, children who experience more severe symptoms at the age of 3.5–6 years may have reduced FA in these neural circuits compared to when they were younger, whereas those with milder symptoms may instead have maintained or even increased their FA. A longitudinal study would clearly be required to confirm this possibility.

A limitation of the current study is its cross-sectional nature and restricted age range (3.5–6 years old). Given that ASD is a neurodevelopmental disorder patterns of structural differences are likely to differ with age and only a longitudinal design study can address this. However, a specific objective of the current study was to establish changes at the age when symptoms have become fully established and diagnosed and where therapeutic interventions are likely to start or already be underway. A second limitation is we did not determine whether observed changes are specific to ASD or might also occur in children with developmental delay, for example. A final limitation is that we could only obtain two small datasets for independent analysis of discrimination accuracy although the findings were very encouraging.

In summary, by employing a fine-grained, brain-wide analysis of structural differences for regional connections in the brains of young (3.5–6 years old) autistic compared with typically developing children we have identified a number of structural connections mainly involving the frontal lobe exhibiting increased FA but negatively associated with symptom severity. Differences in these structural connections show high accuracy (>96%) in discriminating autistic children from TD children which generalizes to discriminating individuals in independent novel datasets. These new findings suggest that differences in structural connections primarily involving the frontal cortex of young autistic children can be applied as a potentially reliable and generalizable biomarker for ASD diagnosis at an individual level and for assessing the efficacy of therapeutic interventions in order to improve the clinical management of individual autistic children for precision medicine.

## Data Availability

Individual participant data and the data dictionary defining each field in the set will not be made available as all participants did not consent to have their data as a public resource. The group-level data results as well as the data processing code which do not disclose the participants’ information will be available with publication from the corresponding authors (KMK and RZ) on reasonable request (including a research proposal), subject to review.
